# Comprehensive Effect of Arc and Ultrasonic Energy on MIG Arc Ultrasonic Welding

**DOI:** 10.3390/ma14174884

**Published:** 2021-08-27

**Authors:** Qihao Chen, Chengcheng Wang, Yihao Wang, Jiahui Wang, Sanbao Lin, Jiayou Wang

**Affiliations:** 1 Provincial Key Laboratory of Advanced Welding Technology, Jiangsu University of Science and Technology, Zhenjiang 212003, China; chengcheng422818@163.com (C.W.); WYH869700@163.com (Y.W.); wjh18852895276@163.com (J.W.); jywang@just.edu.cn (J.W.); 2 State Key Laboratory of Advanced Welding and Joining, Harbin Institute of Technology, Harbin 150001, China; sblin@hit.edu.cn

**Keywords:** ultrasonic, arc, formation, microstructure, mechanical property

## Abstract

Ultrasonic energy is introduced into the Metal Inert Gas (MIG) welding arc and weld pool by superposition of an ultrasonic frequency current. In this study, the arc shape, arc energy, and ultrasonic energy that responded to ultrasonic excitation voltage and frequency is investigated. The comprehensive influence of arc and ultrasonic energy on weld formation, microstructure, and mechanical properties is further studied. The arc and ultrasonic energy are analyzed by using a high-speed camera and microphone, respectively. The results showed that the arc width increased, and the arc energy density decreased after the superposition of ultrasonic current. The arc height could be compressed under certain ultrasonic excitation parameters. The ultrasonic excitation voltage and frequency had a direct influence on the ultrasonic energy. The arc height, arc energy density, and ultrasonic energy together determined the weld width. Ultrasound could effectively refine the microstructure of the weld zone and fusion zone but had little effect on the heat-affected zone. Ultrasound improved the hardness of the joint by refining the grain and the second phase. The joint hardness was the highest when the ultrasonic excitation voltage was 100 V, and the frequency was 30 kHz.

## 1. Introduction

The ultrasonic energy could effectively compress the arc [1,2], promote droplet transition [3], refine weld microstructure [4,5,6], and improve the mechanical properties of the welded joint [7,8]. Ultrasonic has great application potential in the arc welding process. There are two main ultrasonic implementing methods in the welding process. One is in the form of mechanical vibration, and the other is in the form of superimposing ultrasonic frequency current. The arc ultrasonic welding method introduces ultrasonic energy into the welding process in the form of superimposing ultrasonic frequency current [9]. Compared with the mechanical vibration method, the arc ultrasonic welding method offers a better application prospect because it does not change the welding torch structure.

Since the arc ultrasonic welding technology was proposed in 1999, researchers have performed various researches on the equipment and welding effect. At present, the study on arc ultrasonic welding equipment is mainly divided into two directions: (a) integration of welding power supply and ultrasonic excitation power supply; and (b) the welding power supply is relatively independent of the ultrasonic power supply, and the welding power supply and the ultrasonic power supply are connected by the isolation coupling device. The sheet of Ti-6Al-4V was welded by connecting the ultrasonic excitation power supply in parallel with the Tungsten Inert Gas (TIG) welding power supply, and the results showed that the welding arc had broadband response characteristics, the excitation current was the main factor affecting the ultrasonic excitation intensity, and the grain size of the welded joint was refined [10]. Wang established a 3-D plasma-acoustic fully coupled mathematical model to analyze the generation, propagation, and attenuation process of ultrasound in TIG arc. The results show that the ultrasonic wave is actuated by a combination of thermal pressure and Lorentz force from harmonic perturbation of exciting currents. The ultrasonic frequency has a great influence on acoustic power, acoustic absorption, and acoustic streaming [11]. Zhu used self-made isolation coupling devices to connect the TIG power supply and ultrasonic excitation power supply, and analyzed the influence of ultrasonic excitation current and frequency on the pore and strength of the welded joint of MGH956 alloy. The research showed that the porosity decreased significantly, and the strength was improved obviously when the ultrasonic excitation current increased to 20 A or 30 A. Porosity and tensile strength improved when the ultrasonic excitation frequency decreased from 60 kHz to 30 kHz [12]. Wang welded Inconel 718 super-alloy by the parallel connection of DC TIG power supply and ultrasonic power supply. The results showed that specific ultrasonic excitation frequency could improve the tensile strength and plasticity of the welded joint and increase the weld depth to width ratio [13]. Lei also welded SiCp/Al MMCs and SiCp/6061Al materials by parallel of TIG welding power supply and ultrasonic power supply. The results showed that appropriate ultrasonic excitation frequency could fine the microstructure, enhance the distribution of the reinforcement phase, and improve the tensile strength [14,15]. The welding experiment of CLAM steel shows that the arc-ultrasonic technique effectively refines the microstructure, provides even distribution of carbides, and improves the mechanical performance of the weld metal and the heat-affected zone (HAZ) [16]. The TIG welding power supply and the ultrasonic excitation power supply were coupled by the parallel connection of two power supplies. The BT20 titanium alloy material was welded, and the welding wire was TC3. The ultrasonic excitation frequency range was from 20 kHz to 500 kHz, and the ultrasonic excitation current was about 10 A. The results showed that the arc-ultrasonic energy refined the grain structure in the weld metal and improved the fatigue life of welded joint [17]. Yang built an integrated power supply-ultrahigh frequency pulsed gas tungsten arc welding (UHFP-GTAW), and the relationship between electromagnetic force and pulse current was studied [18]. The results showed that the pulse frequency affects the electromagnetic force. The axial electromagnetic force decreases with the increase in the pulse frequency. The radial electromagnetic force is the key for the arc constriction and it reduces with decreasing root radius, which pushes the arc plasma to steady state. Anna applied the welding power in parallel with the ultrasonic excitation power in the submerged arc welding process to study the influence of the ultrasonic excitation frequency and current on the weld formation, and the research showed that the weld formation had no significant change under the set ultrasonic excitation parameters [19]. Zhang investigated the relationship between the ultrasonic excitation frequency and the ultrasonic excitation energy, and discussed the resonance phenomenon between the arc and ultrasonic [9]. The results showed that the resonance frequency is decided by the frequency of the external electrical signal and the weld appearance. Huang developed high-frequency TIG welding equipment and analyzed its arc shape. The study showed that the arc expansion speed is greater than the contraction speed when the pulse current changes abruptly [20]. Zhao’s research results show that the pulsed TIG arc voltage increases with the increase in pulse frequency [21].

From the above summary, it can be seen that the researchers mainly conducted in-depth studies on TIG arc ultrasonic, but less on MIG arc ultrasonic. The characteristics of the MIG arc are different from those of the TIG arc. The MIG arc is unstable, and the arc height changes dynamically due to the droplet transition. Therefore, the ultrasonic excitation characteristics of the MIG arc will be different from that of the TIG arc. In addition, MIG welding is more efficient and widely used than TIG welding. Therefore, it is necessary to investigate the characteristics of arc ultrasonic in MIG welding. Li studied the acoustic-electric characteristics of ultrasonic arc MIG welding of 6061 aluminum alloy and preliminarily analyzed the relationship between ultrasonic excitation energy and ultrasonic excitation parameters, the weld formation, and microstructure changes [22], but did not make a specific analysis of the MIG arc morphology under the influence of ultrasonic current superposition.

Aiming at the aluminum alloy MIG arc ultrasonic welding process, this paper investigates the effect of ultrasonic current superposition on arc and ultrasonic excitation energy, and investigates the change mechanism of weld formation, microstructure, and mechanical properties by comprehensively considers the arc characteristics and ultrasonic effect.

## 2. Materials and Methods

### 2.1. Materials

The base was is a 4.0-mm-thick 5083 aluminum alloy with the chemical composition (wt pct: ≤0.40 Si, ≤0.1 Cu, 4.0–4.9 Mg, ≤0.25 Zn, 0.4–1.0 Mn, ≤0.15 Ti, 0.05–0.25 Cr, and ≤0.40 Fe). In this study, all the welding experiments were bead-on-plate welding. The welding wire of ER 5183 (wt pct: 4.7–5.2 Mg, 0.20 Cr, 0.15 Fe, ≤0.05 Cu, 0.10 Zn, 0.05 Mn, and 0.10 Ti) with a diameter of 1.2 mm was employed. The size of the workpiece to be welded was 200 mm × 100 mm. Polishing and cleaning the base material surface with sandpaper and alcohol was carried out before welding.

### 2.2. Experimental Set-Up and Methods

**Figure 1:**
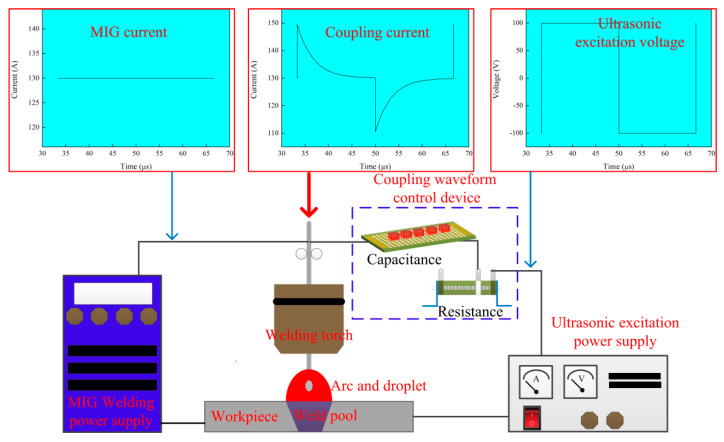
Schematic diagram of MIG arc ultrasonic welding system.

The schematic diagram of the MIG arc ultrasonic welding system is shown in Figure 1. The welding torch was fixed above the workpiece during welding, while the workpiece moved with a constant welding speed of 7.93 mm/s. The setup included a welding system and an ultrasonic coupling system. The welding system mainly consisted of a MIG welding power supply and welding torch. The ultrasonic coupling system mainly consisted of an ultrasonic excitation power supply and a coupling waveform control device.

The coupling waveform control device consists of the capacitor and the resistance. It could change waveform of the ultrasonic frequency current by controlling the capacitor charge-discharge time. In this study, an ultrasonic frequency current waveform with low additional heating input was applied to the welding experiment. 

An AOTAI Pulse MIG 350 welding power (Aotai Electric Co., Ltd., Jinan, China) supply was applied in a direct current electrode negative condition. In the study, the welding current, the welding voltage, and welding velocity were constant. The welding current was 130 A and the welding voltage was about 21.9 V. The shielding gas was argon of 99.99% purity, and the airflow was 15 L/min. The ultrasonic excitation power supply could generate an ultrasonic frequency, alternating rectangular electrical signal. The ultrasonic excitation voltage could be adjusted from 0 V to 200 V. The ultrasonic excitation frequency could be adjusted at the range from 15 kHz to 80 kHz. In the study, the output voltage and frequency of the ultrasonic excitation power supply were changed, as shown in Table 1. The welding experiments were carried out three times for every experimental parameter.

The ultrasonic frequency current was coupled with the MIG welding current, resulting in a ultrasonic frequency variation of welding current and arc force. The arc force with ultrasonic frequency could act as an ultrasonic emission source, which generates ultrasonic vibration acting on the arc and the weld pool.

The acoustic field around the arc was measured by the sensor, which is a 4939 air-free-field microphone from B&K Company (Skodsborgvej, Denmark). The distance between the sensor and the arc center was 60 cm. The acoustic energy of the ultrasonic signal and the ordinary arc signal were measured, respectively. 

A high-speed video camera (CamRecord3000X2, Optronis Co., Kehl, Germany) was used to film the arc shape and analyze the arc brightness. A laser acts as the backlight. The parameters of the high-speed camera were a sample rate of 4677 fps and exposure time of 200 μs.

Three cross-sectional samples of each welded profile were equidistantly cut by electro-discharge machining from the cross-section of the 5083 Al weld beads. The weld width and area were measured to characterize the weld formation by an optical microscope (Smart zoom 5, ZEISS Co., Oberkochen, Germany) and Image-Pro Plus software (v 6.0). The grain orientation and size in the transverse cross-section of the welded joint were analyzed by EBSD technology. The second phase of the welded joint was characterized by SEM (JSM-7900f, JEOL, Tokyo, Japan) and EDS (Octane Elect EDS, EDAX, Pleasanton, CA, USA). The calculation method of the second phase area in the welded joint is as follows. Three experimental samples were prepared for each experimental parameter. The weld center zone, fusion zone, and heat-affected zone of each sample were photographed by the scanning electron microscope. The Image-pro Plus software was used to identify the contour of the second phase in the photos, and the second phase was labeled. The statistical function of the software was used to obtain the area of the second phase in each region, and then to record the average. Three images were measured for each region, and the average value of the second phase area was taken. The transverse and longitudinal hardness distribution in the welded joint was measured through a hardness tester.

## 3. Results

### 3.1. Arc Behavior and Weld Formation 

**Figure 2:**
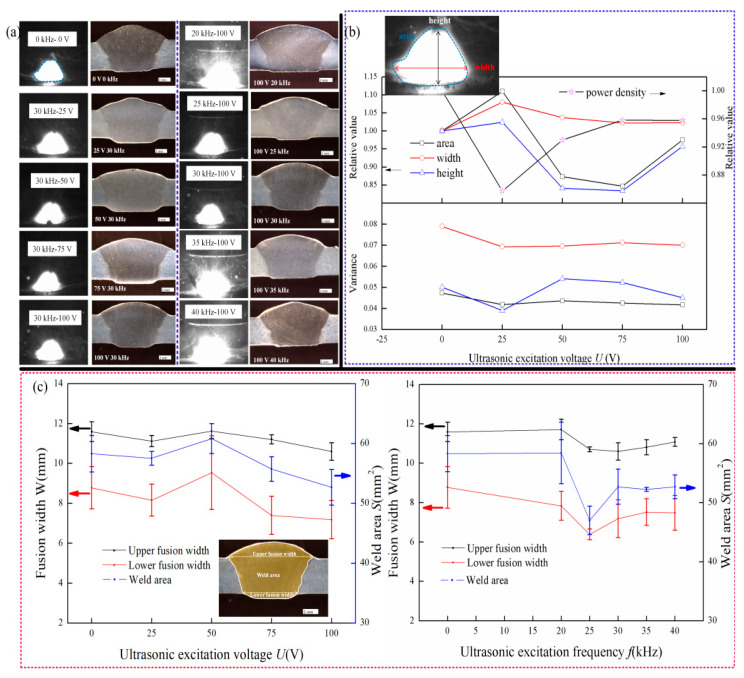
(**a**) Morphologies of the welding arc and weld formation, and (**b**) characteristics of welding arc, and (**c**) characteristics of fusion width and weld area.

The coupling of MIG welding current with ultrasonic frequency pulse current generates ultrasonic energy and an additional heating input, which could change the arc shape and energy. The effect of ultrasonic frequency pulse current on the arc behavior and weld formation was investigated, as shown in Figure 2. Figure 2a shows the morphologies of the welding arc and weld formation under the different ultrasonic excitation voltage and current. Figure 2b shows the characteristics of welding arc at the different ultrasonic excitation voltage. Figure 2c shows the characteristics of fusion width and weld area at the different ultrasonic excitation voltage and current. The ultrasonic excitation frequency has an obvious influence on the arc brightness compared with the ultrasonic excitation voltage. The arc brightness increases obviously when the ultrasonic excitation frequency is 20, 35, and 40 kHz. Arc brightness reflects the arc energy. The greater the arc brightness, the greater the arc energy.

The arc width, height, area, and energy density with different ultrasonic excitation voltages were measured. The effect of ultrasonic excitation frequency on arc shape is not analyzed because the brighter arc affects the measurement of an arc shape. As Formula (1) shows, the arc energy density is related to the arc energy and arc area.

(1) $$Q=\frac{P}{S}$$

where *Q* is the arc energy density, *P* is the arc power, and *S* is the arc area calculated according to the arc width from Figure 2a.

As given in Formula (2), the arc power consists of the original arc power and the additional heat input power.

(2) $$P=P_{1}+P_{2}$$

where *P*_1_ is the original arc power, *P*_2_ is the additional heat input power. 

**Figure 3:**
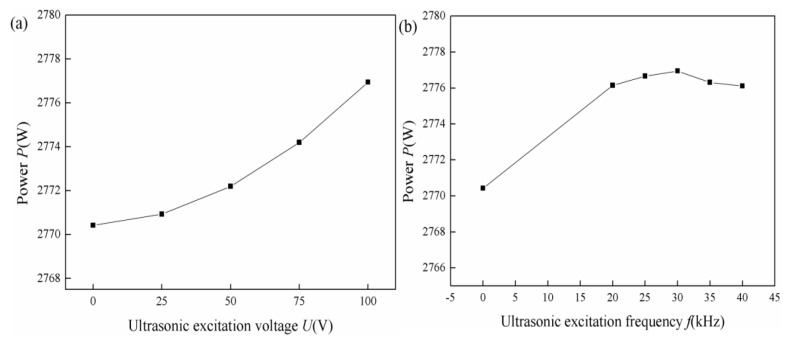
Arc power with different ultrasonic excitation parameters: (**a**) ultrasonic excitation voltage; (**b**) ultrasonic excitation frequency.

The current had a direct effect on arc power, which is given in Formula (3)

(3) $$P=\frac{\int_{0}^{t}I^{2}rdt}{t}$$

where *I* is the coupling current after coupling of the ultrasonic frequency pulse current with the MIG welding current, and the coupling current is obtained by the software NI Multisim (v 14.0). r is the arc resistance. In this study, the coupling waveform control resistance is equal to 10 Ω; the arc resistance is equal to 0.2 Ω; and the capacitance is equal to 0.33 μF. The equivalent circuit was established, and the coupling welding current was simulated by NI Multisim software. The arc energy was calculated by Formula (3). As shown in Figure 3, the arc power gradually increases with the increase in the ultrasonic excitation voltage. The arc power increases first and then stays constant gradually with the increase in the ultrasonic excitation frequency.

The arc width, height, area, and energy density with different ultrasonic excitation voltages are shown in Figure 2b. The change in the arc area and arc height is similar. The relationship between the arc height, area, and the ultrasonic excitation voltage is nonlinear. The arc area and height first increases, then decreases, and then increases with the increase in the ultrasonic excitation voltage. The arc width increases first and then decreases with the increase in the ultrasonic excitation voltage. However, the arc is always wider than that without the ultrasonic excitation. The calculation results of arc energy density show that the arc energy density decreases first and increases with the increase in ultrasonic excitation voltage. However, the energy density is less than the arc energy density without ultrasonic excitation.

All welds are full penetration. The upper weld width, lower weld width, and melting area for weld were analyzed statistically. It can be seen from Figure 2c that the change in weld width and weld area shows volatility as the ultrasonic excitation voltage variation. The weld width and weld area are similar to the weld characteristics without ultrasonic excitation when the ultrasonic excitation voltage is 25 V. The weld width and weld area is the maximum when the ultrasonic excitation voltage is 50 V. The weld width and weld area decrease gradually when ultrasonic excitation voltage exceeds 50 V. By changing the ultrasonic excitation frequency and fixing the ultrasonic excitation voltage, the weld width and weld area are smaller when the ultrasonic excitation frequency is around 25 kHz and 30 kHz. However, the characteristics of other ultrasonic excitation frequencies are similar to those without ultrasonic excitation.

### 3.2. Ultrasonic Energy

**Figure 4:**
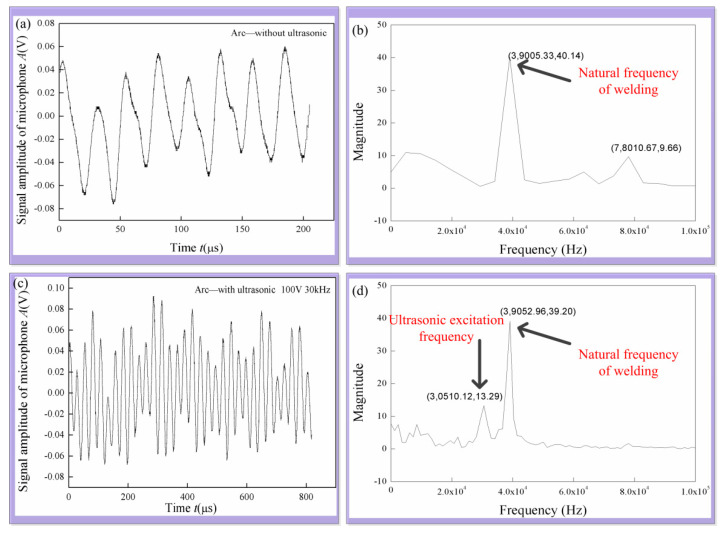
Acoustic signal detection around the arc: (**a**) original MIG, time-domain signal; (**b**) original MIG, frequency-domain signal; (**c**) 30 kHz ultrasonic coupling, time-domain signal; (**d**) 30kHz ultrasonic coupling, frequency domain signal.

The ultrasonic vibration could be generated by the arc, and the ultrasonic intensity was measured with a microphone to obtain the time domain signal. The Fourier transform of the time domain signal was performed to obtain the frequency domain signal. As shown in Figure 4, the frequency domain signal includes two main frequencies, one frequency is the ultrasonic excitation frequency applied, and the other frequency is about 39 kHz, which is the natural frequency of MIG welding equipment.

**Figure 5:**
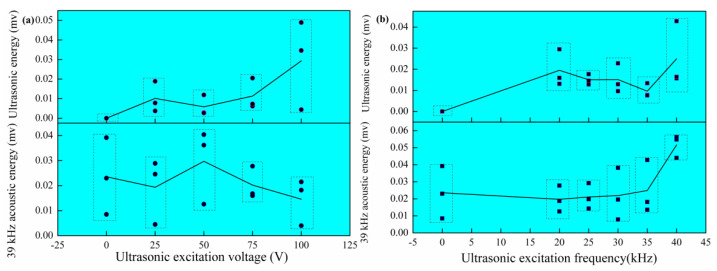
The acoustic energy with different ultrasonic excitation: (**a**) ultrasonic excitation voltage; (**b**) ultrasonic excitation frequency.

The influence of ultrasonic excitation voltage and frequency on the acoustic energy was analyzed, as shown in Figure 5. As a whole, the ultrasonic intensity increases gradually with the increase in ultrasonic excitation voltage. The ultrasonic intensity decreased with the increase in ultrasonic excitation frequency. However, the ultrasonic intensity increases significantly when the ultrasonic excitation frequency is about 40 kHz. The acoustic intensity of 39 kHz has no relationship with the ultrasonic excitation. When the ultrasonic excitation frequency is 40 kHz, the acoustic intensity is significantly enhanced.

### 3.3. Microstructure 

**Figure 6:**
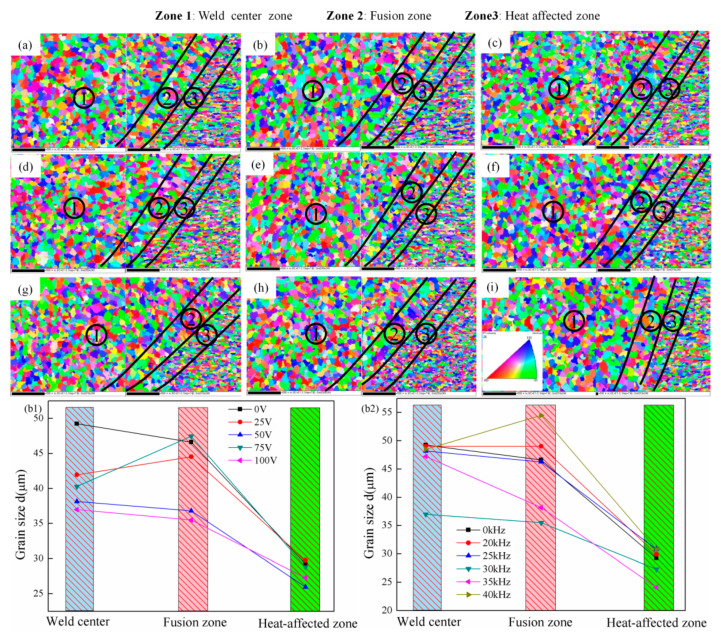
Grain orientation at different ultrasonic excitation parameters from (**a**) 0 V, 0 kHz, (**b**) 25 V, 30 kHz, (**c**) 50 V, 30 kHz, (**d**) 75 V, 30 kHz, (**e**) 100 V, 30 kHz, (**f**) 100 V, 20 kHz, (**g**) 100 V, 25 kHz, (**h**) 100 V, 35 kHz, (**i**) 100 V, 40 kHz; and Grain size from different: (**b1**) ultrasonic excitation voltages, and (**b2**) ultrasonic excitation frequency.

The influence of the ultrasonic excitation voltage and frequency on the grain orientation and size in the MIG welded joint of 5083 aluminum alloy is shown in Figure 6. The Figure 6a–i are the grain orientation at the different ultrasonic excitation voltage and frequency. The weld grains without ultrasonic action are equiaxed grains with random grain orientation. The weld grain is still equiaxed grain with random orientation after implementing the ultrasonic excitation. 

The equivalent diameters of grains in the weld center zone, the fusion zone, and the heat-affected zone were statistically analyzed. The ultrasonic excitation has a larger effect on grain refinement in the weld zone and the fusion zone, but a smaller effect on grain refinement in the heat-affected zone. Fixing the ultrasonic excitation frequency and adjusting the ultrasonic excitation voltage and the grain size in the three regions of the welded joint is the smallest when the ultrasonic excitation voltage is 50 V and 100 V. The effect of ultrasonic excitation frequency on grain refinement is different from that of ultrasonic excitation voltage. The grain size in the welded joint decreases when implementing the specific ultrasonic excitation frequency. The grain size in the three regions of the welded joint is the smallest when the ultrasonic excitation frequency is 30 kHz and 35 kHz.

The grains size increase in the fusion zone and heat-affected zone and exceed that without ultrasonic excitation when the ultrasonic excitation frequency is 20 kHz and 40 kHz. The grain coarsening is very serious in the fusion zone when the ultrasonic excitation frequency is 40 kHz. 

**Figure 7:**
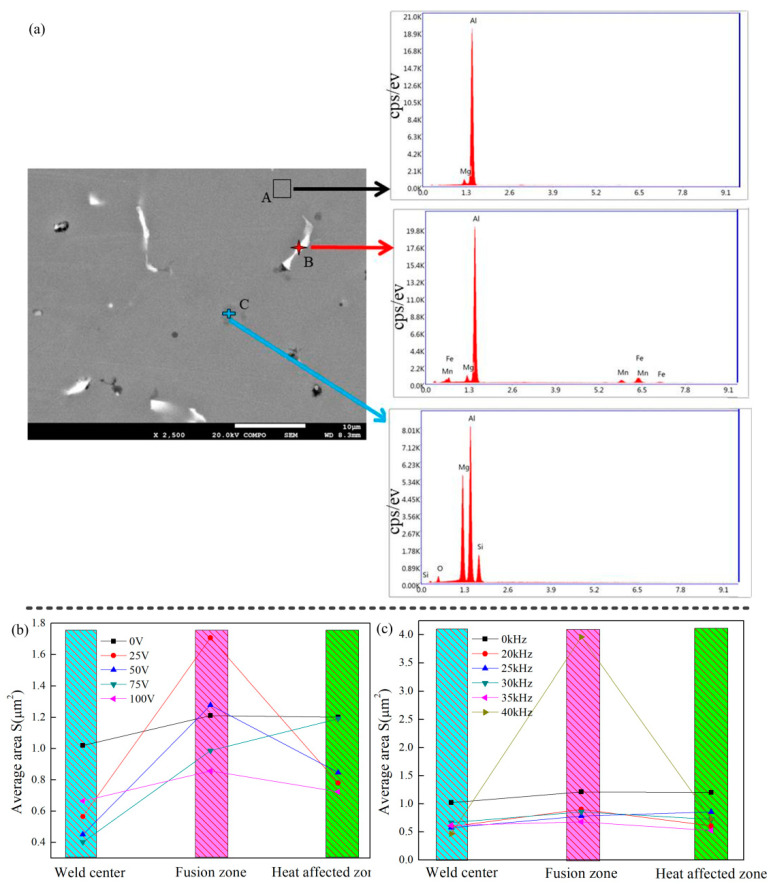
(**a**) SEM and EDS analysis on the second phase composition; The second phase size from different (**b**) ultrasonic excitation voltage, and (**c**) ultrasonic excitation frequency.

SEM and EDS were used to analyze the second phase of the welded joint. As shown in Figure 7, the matrix is an Mg-containing aluminum-based solid solution, and the second phase consists of two categories. The first kind of phase mainly contains Al, Mg, Mn, and Fe elements, and this phase is white and has a large content in the joint. The second phase mainly contains Al, Mg, and Si elements, which is grey and has less content. Therefore, this study investigated the size distribution of the first kind phase.

As shown in Figure 7, the arc ultrasonic has a significant refinement effect for the second phase in the weld zone and fusion zone. The ultrasonic-assisted refinement ability for the second phase is weak in the heat-affected zone. The effect of ultrasonic excitation voltage on grain refinement is greater than that of the ultrasonic excitation frequency. The effect of ultrasonic-assisted refinement on the second phase and grain is non-synchronous. Fixing the ultrasonic excitation frequency and adjusting the ultrasonic excitation voltage. The size of the second phase is coarse in the fusion zone when the ultrasonic excitation parameter is 25 V and 40 kHz.

### 3.4. Hardness 

**Figure 8:**
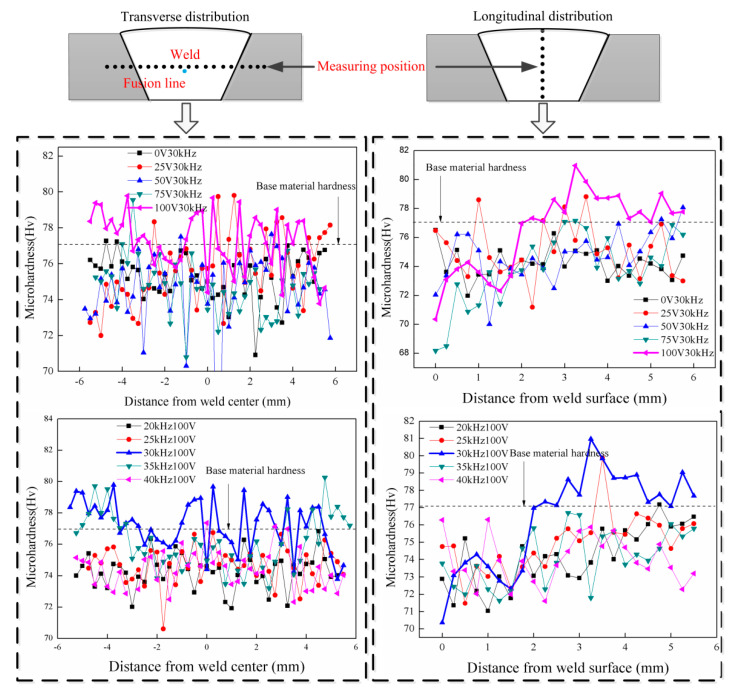
The hardness of welded joint with different ultrasonic excitation voltages and frequencies.

As can be seen from Figure 8, the transverse and longitudinal hardness of the welded joint was measured under different ultrasonic excitation voltages and frequencies.The hardness of welded joint increases significantly, and the hardness in some positions of the weld is greater than that of the base material when the ultrasonic excitation voltage is 100 V and the ultrasonic excitation frequency is 30 kHz. The hardness of the weld gradually increases from the weld surface to the bottom of the weld.

## 4. Discussion

### 4.1. Mechanism of Weld Width Variation 

**Figure 9:**
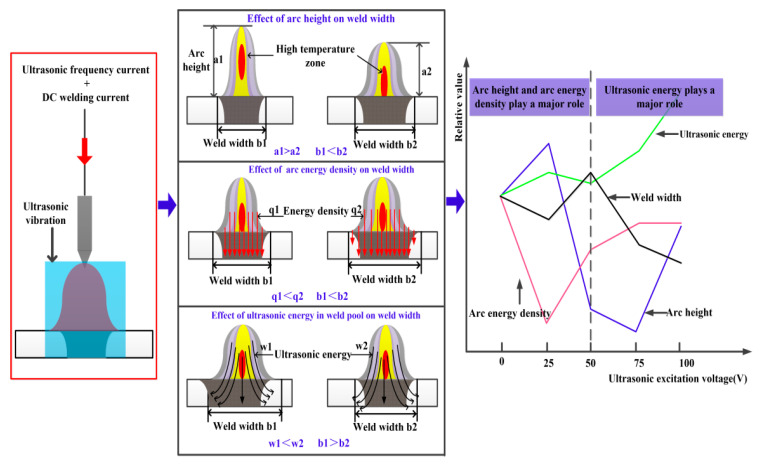
Diagram of influence of arc ultrasonic excitation voltage on weld width.

The influence mechanism of arc ultrasonic excitation voltage on weld width is shown in Figure 9. The coupling of ultrasonic frequency pulse current and DC MIG welding current could change the welding current waveform and generate ultrasonic energy, which acts on the weld pool. Thus, the arc shape and energy density are different from the original welding characteristic. By comprehensive analysis of the experimental results in Figure 2 and Figure 5, it is concluded that the arc energy density, arc height, and ultrasonic energy have a direct influence on weld width. As shown in Figure 9, the weld width increases with the increase in arc energy density when the arc height and ultrasonic energy are constant. The weld width increases with the decrease in arc height when the arc energy density and ultrasonic energy are constant. Previous studies have shown that the weld width decreases when the ultrasonic energy acts on the weld pool [23].

The ultrasonic energy is small in the weld pool, and the weld width is mainly affected by the arc height and arc energy density when the ultrasonic excitation voltage is less than 50 V. The arc energy density decreases, and the arc height increases when the ultrasonic excitation voltage increase from 0 V to 25 V, resulting in the decrease in the weld width. The arc height decreased significantly when the ultrasonic excitation voltage is 50 V, leading to the weld pool closer to the high-temperature zone of the arc, as shown in Figure 9. Meanwhile, the arc energy density increases, leading to an increase in weld width. The arc energy density is almost constant, and the arc height and ultrasonic energy gradually increase when the ultrasonic excitation voltage exceeds 50 V, resulting in a decrease in weld width.

### 4.2. Mechanism of Microstructure Refinement 

**Figure 10:**
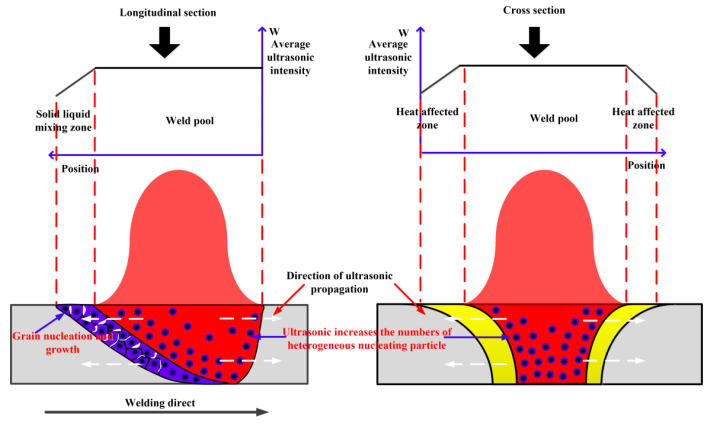
Diagram of arc-ultrasonic assisted microstructure refinement mechanism.

The mechanism of welded joint microstructure refinement is shown in Figure 10. The ultrasonic vibration comes from the ultrasonic frequency variation of the arc force. The arc force has a greater effect on liquid fluid than the solid, so the ultrasonic energy has a big effect on the weld zone and fusion zone. Aluminum alloy contains oxide and carbide particles with a high melting point, which are difficult to melt in an arc welding pool. These refractory particles could be used as heterogeneous nucleating particles for grain nucleation in the weld pool [24]. The ultrasonic vibration could generate a cavitation phenomenon in the weld pool when the ultrasonic pressure exceeds the cavitation threshold. Cavitation will produce high temperature, high pressure, and acoustic jet, and the cavitation improves the heterogeneous nucleation rate [25]. The cavitation could promote partial solidification of aluminum alloy melt on the surface of particles, which leads to an increase in the size of the refractory particles. More of the particles are larger than the critical nucleation radius and can be used for effective nucleation during the crystallization of the weld pool, which increases the nucleation rate of the grains and refines the weld grains. The number of heterogeneous nucleating particles is increased by ultrasonic in the weld pool. Thus, the rate of heterogeneous nucleation is improved. The heterogeneous grains nucleate and crystallize in the solid–liquid mixing zone. The equiaxed crystal could limit the growth of the second phase. Therefore, the effect of ultrasound on microstructure refinement of the fusion zone and weld zone is more obvious than that of the heat-affected zone. The ultrasonic vibration could propagate into the heat-affected zone through the solid–liquid boundary. More ultrasonic energy attenuates when the ultrasonic vibration passes through the solid–liquid interface, which results in less vibration energy in the heat-affected zone to refine the microstructure.

### 4.3. Hardness Improvement Mechanism

According to the strengthening theory, the mechanical properties of the welded joint can be improved by control the distribution of grain and the second phase. 

According to the Hall-Petch relation,

(4) σ=σ0+kd^(−12)

where *σ*_0_ is the yield strength of a single crystal, *σ* is the total yield strength, *k* is a constant, and *d* is the grain size. 

The yield strength and hardness of welded joint could be improved when the grain size decreases. According to the second phase strengthening theory, the strength could be improved when the second phase of small size is evenly dispersed in the matrix phase. The smaller the size of the second phase and the more even the distribution, the better the performance of the welded joint. Therefore, the joint hardness is the aggregate effect of grain size and the second phase size. The grain size and the second phase size reach the best match, and the hardness is the maximum when the ultrasonic excitation voltage is 100 V and the frequency is 30 kHz.

## 5. Conclusions

1. The coupling of ultrasonic frequency pulse current and DC MIG welding current could change the arc shape and arc energy density, and generate ultrasonic energy. The arc width increases after the ultrasonic coupling. Certain ultrasonic excitation parameters could compress the arc height. The ultrasonic energy increases with the increase in the ultrasonic excitation voltage. The arc energy density, arc height, and ultrasonic energy determine the weld width. The weld width decreases gradually when the ultrasonic excitation voltage exceeds 50 V.

2. Ultrasonic excitation has a good refining ability on the grain and the second phase in the weld zone and the fusion zone. However, it has no significant effect on the heat-affected zone. It is not conducive to microstructure refinement when the ultrasonic excitation frequency is close to the natural frequency of MIG welding equipment.

3. Ultrasonic coupling can improve the hardness of welded joints. The hardness reaches the maximum when the ultrasonic excitation frequency is 30 kHz and ultrasonic excitation voltage is 100 V. The improvement of hardness is the result of grain refinement and second phase refinement.

## Figures and Tables

**Table 1:** Ultrasonic excitation parameters.

N_O_	1	2	3	4	5	6	7	8	9
Excitation voltage (V)	0	25	50	75	100	100	100	100	100
Excitation frequency (kHz)	0	30	30	30	30	20	25	35	40

## Data Availability

The data presented in this study are available on request from the corresponding author.
